# Minimising Mortality in Endangered Raptors Due to Power Lines: The Importance of Spatial Aggregation to Optimize the Application of Mitigation Measures

**DOI:** 10.1371/journal.pone.0028212

**Published:** 2011-11-28

**Authors:** Francisco Guil, Mariana Fernández-Olalla, Rubén Moreno-Opo, Ignacio Mosqueda, María Elena Gómez, Antonio Aranda, Ángel Arredondo, José Guzmán, Javier Oria, Luis Mariano González, Antoni Margalida

**Affiliations:** 1 CBD-Habitat Foundation, Madrid, Spain; 2 Tragsatec, Gerencia de Biodiversidad, Madrid, Spain; 3 E.T.S. Ingenieros de Montes, Universidad Politécnica de Madrid, Madrid, Spain; 4 Dirección General de Montes y Espacios Naturales, Junta de Comunidades de Castilla-La Mancha, Toledo, Spain; 5 Servicio de Especies Amenazadas, Dirección General de Medio Natural y Política Forestal, Spanish Ministry of the Environment and Rural and Marine Affairs, Madrid, Spain; 6 Bearded Vulture Study and Protection Group, El Pont de Suert, Lleida, Spain; University of Bern, Switzerland

## Abstract

Electrocution by power lines is one of the main causes of non-natural mortality in birds of prey. In an area in central Spain, we surveyed 6304 pylons from 333 power lines to determine electrocution rates, environmental and design factors that may influence electrocution and the efficacy of mitigation measures used to minimise electrocution cases. A total of 952 electrocuted raptors, representing 14 different species, were observed. Electrocuted raptors were concentrated in certain areas and the environmental factors associated with increased electrocution events were: greater numbers of prey animals; greater vegetation cover; and shorter distance to roads. The structural elements associated with electrocutions were shorter strings of insulators, one or more phases over the crossarm, cross-shaped design and pylon function. Of the 952 carcasses found, 148 were eagles, including golden eagle (*Aquila chrysaetos*), Spanish imperial eagle (*Aquila adalberti*) and Bonelli's eagle (*Aquila fasciata*). Electrocuted eagles were clustered in smaller areas than other electrocuted raptors. The factors associated with increased eagle electrocution events were: pylons function, shorter strings of insulators, higher slopes surrounding the pylon, and more numerous potential prey animals. Pylons with increased string of insulators had lower raptor electrocution rates than unimproved pylons, although this technique was unsuccessful for eagles. Pylons with cable insulation showed higher electrocution rates than unimproved pylons, both for raptors and eagles, despite this is the most widely used and recommended mitigation measure in several countries. To optimize the application of mitigation measures, our results recommend the substitution of pin-type insulators to suspended ones and elongating the strings of insulators.

## Introduction

Electrocution has been considered one of the most significant causes of mortality among raptors [1]–[4]. This type of mortality affects raptor population dynamics by regulating the density of the birds [5], targeting specific age classes and changing populations [6]. Thus, this non-natural cause of mortality is capable of destabilising populations [7] and could potentially cause local extinctions [8].

The Iberian Peninsula has one of the highest raptor densities in the western Paleartic, particularly of large eagles (genus *Aquila*) [9]. Although electrocution is one of the main causes of mortality for these species [2], [3], few broad studies have been undertaken on the factors influencing electrocution on eagles (although see [10]). Many existing studies evaluate birds' interaction with power lines in local areas [11], or focus on a single type of pylon [12]. However, because each pylon has structural characteristics, such as the kind of insulators present and the function of the pylon, these characteristics could influence mortality rates [13]. In this sense, it has been documented than both the insulators (pin-type or suspended, [10], [11]) and the number of phases above the crossarm [14], have an impact on the probability of birds being killed by electrocution.

For raptors, it has been said that this mortality corresponds to ‘contagious’ phenomena, in other words, concentrated in terms of space [15], [16], although there is little proof of these phenomena [11], [16]. There is a need to understand the spatial distribution of the mortality across extensive areas, in order to develop strategies that can reduce this phenomenon.

In Spain, in recent years, more than 25 million Euros have been spent to reduce the impact of power lines on raptors [17]–[19]. Those measures where mainly directed toward the recovery of the Spanish imperial eagle (*Aquila adalberti*). Where corrections have been implemented, the recovery of the Spanish imperial eagle has been remarkable, [7], [17], although this might be due to other factors [2]. However, other species highly susceptible to electrocution, such as the threatened Bonelli's eagles (*Aquila fasciata*) or golden eagles (*Aquila chrysaetos*), have slightly decreased in population or have maintained their numbers [20], [21]. In addition, the long-term efficiency of these measures is unknown [12]. Therefore, it is important to determine the efficiency of these measures in a pylon-per-pylon approach.

This study addresses the abovementioned research gap by focusing on mitigation measures, environmental, spatial and structural factors that influence the electrocution of specific bird groups, such as large eagles, over a large geographic area. Our goals are to describe raptor mortality caused by electrocution in a large area in central Spain, being the objectives of this study: 1) determine whether mortality events are distributed evenly over all power lines or are concentrated around certain lines; 2) analyse structural and environmental characteristics to determine what influences raptor electrocution rates, particularly for *Aquila* genus (henceforth eagles); and 3) examine the efficiency of mitigation measures implemented in this area prior to this study.

## Materials and Methods

### Ethics Statement

All the work was conducted in accordance with relevant national and international guidelines, and conforms to the legal requirements of the regional governments and Public Administration.

### Study area

The study area encompasses the provinces of Ciudad Real and Albacete in south-eastern Spain, with 20 479 km^2^. The study area contains abundant prey for raptors, including wild rabbits (*Oryctolagus cuniculus*) and red-legged partridges (*Alectoris rufa*) [22], [23]. Vegetation is characterised by holm oak (*Quercus ilex*), shrubs such as *Quercus coccifera, Cistus ladanifer*, and *Cistus monspeliensis*, and *Stipa tenacissima* (tussock grass) in grazing areas.

Besides being an important habitat for a significant number of raptor species, 20 different raptor species breed in this area, including the endangered cinereous vulture (*Aegypius monachus*), the Egyptian vulture (*Neophron percnopterus*), the Spanish imperial eagle, and the Bonelli's eagle [24], [25].

### Power line survey

**Figure 1:**
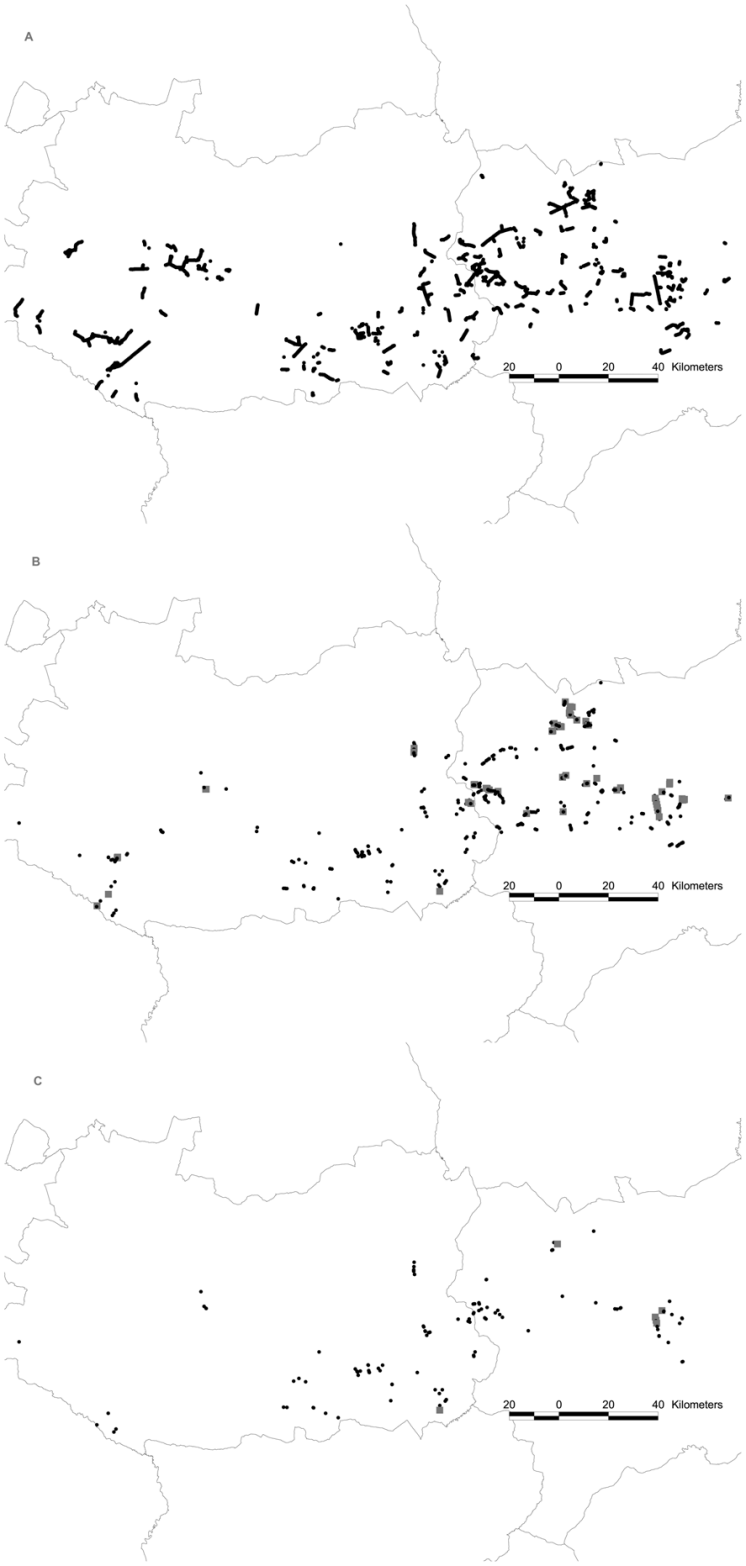
Map of the study area with the pylons surveyed (a), raptor (b) and eagle (c) mortality per pylon. For mortality, black circles indicate 1 or 2 carcasses and grey squares 3 or more.

Between October 2004 and December 2009, 333 power lines (12–66 kV) and 6304 pylons were surveyed on foot, representing 10% of all power line length in this area (Figure 1). Pylons were only surveyed once and the power lines were chosen according to their potential to impact local birds of prey, following the criteria of [10], [11]. We selected preferably power lines with pin-type insulators, pylons with phases over the crossarm or with short strings of insulators (see Supporting Information Figures S1, S2, S3, S4 and S5). Moreover, lines were selected that passed through an environment with open natural vegetation or a scrubland-crop interface.

Almost all power lines were completely surveyed. In some cases, pylons located less than 300 m from human settlements were not surveyed according to [13] (but see [26]–[28]). Electrocuted birds were collected in a 25 m radius around each pylon [11] and identified by species. A record was made for each pylon that included its characteristics and location (UTM) (Table 1) according to methods used in previous studies [10], [11], [13], [29]. As most of the pylon crossarms were made of metal (*n*  = 6231) and only a few of concrete (*n*  = 73) this variable was excluded from analysis. All the pylons were ground wired in the study area.

**Table 1:** Characteristic variables and pylon structure and line recorded for each pylon and line reviewed (for more details see Supporting information).

Structural Variable		Description	N
Function	Holder	Pylons that support the conductors	4727
	Anchor	Pylons that employ horizontal structures to generate cable tension	812
	Special	Pylons that have a special function, such as line intersections, cut-outs, transformers, etc.	765
Model	Flat	All phases are at the same level	2355
	Cross-shaped	Central phase above lateral phase	1475
	Vault	Central phase above lateral phase, always with suspended insulators	2097
	Lattice vault	Central phase above lateral phase, always with suspended insulators, wider than vaults	198
	Three levels	Each phase at one different level	179
Insulators	0	Pin-type insulators	2506
	2–9	Number of insulators at each phase	-
Phases over the crossarm	0,1 or 3	Number of phases over the crossarm	-
Tower	Steel	Tower composition material	3990
	Concrete		2314
Crossarm	Steel	Crossarm composition material	6231
	Concrete		73
Mitigation measures	None	No mitigation measure	5597
	Cable covers	Installation of rubber cable covers	442
	Silicone covers	Installation of silicone cable covers	77
	Insulator substitution	Changing ceramic insulators to glass ones	30
	Extension	Non-conductive steel extension used to elongate the length of the string of insulators in anchor or special pylons	158

Based on previous studies in nearby areas [13], the civil service responsible for oversight has implemented mitigation measures such as the use of insulating rubber or silicone cable covers, changing ceramic insulators to glass ones and string of insulator extensions [19]. The insulation of cables, both with rubber and silicone covers, consisted of the installation of wire covers approximately 1 m to either side of the pylon, in addition to on the strained wire when it was present. This avoids any bird perching on the crossarm and coming into contact with the cable, causing a difference in the electrical charge. Changing ceramic insulators to glass ones consisted of changing the type of insulators, so that greater distances are usually achieved between the crossarm and the wire. Finally, the string of insulator extension consisted in the installation of a non-conductive steel extension used to elongate the length of the string of insulators in anchor or special pylons. Most of these measures were implemented in the 1990s [19]. Therefore, in order to try to assess their efficiency after over 10 years, these measures will be considered another factor to be included in the analyses.

### Environmental variables

To obtain environmental variables surrounding each pylon, the UTM location of each was recorded on a digital map scaled at 1∶50.000 using ArcView 3.1 GIS. Vegetation maps were obtained from the Spanish Ministry of the Environment [30], [31]. Environmental variables were chosen according to whether they influenced electrocution rates (Table 2) based on methods used in previous studies [11], [32]. Topography can affect mortality, if we take into account the fact that raptors prefer exposed high perches. Thus, it likely that the pylons that stand out most on the land will cause the highest mortality rates [4], [11].

**Table 2:** Description of chosen environmental variables.

Environmental Variable	Description
Distance to roads (m)	Distance in meters to paved roads
Distance to paths (m)	Distance in meters to unpaved tracks or paths
Distance to inhabited places (m)	Distance in meters to inhabited places
Bushes (%)	Percentage of surface covered with bushes 25 m around the pylon
Trees (%)	Percentage of surface covered with trees 25 m around the pylon
Slope (%)	Average slope, in%, 25 m around the pylon
Prey abundance	Abundance of prey, in 5 categories, 25 m around the pylon
	0. No prey saw or signs observed
	1. Few signs observed
	2. A single prey observed or presence of several signs
	3. Several preys and presence of abundant signs observed
	4. Many preys and very abundant signs observed

The “distance” variables were obtained from the Nearest Feature V.3.8. extension for ArcView 3.1 [33]. We considered distances to three elements (roads, paths and urban settlements). The remaining parameters were assessed within a 25 m area surrounding each pylon.

Prey abundance (wild rabbit and red-legged partridge) were characterised according to [23]. Thus, five abundance categories were used, based on direct observations and the abundance of tracks observed when looking for carcasses in a 25-m radius around each pole (see Table 2).

After preliminary analyses, we chose to use categorical distance variables (distance to road, paths and human settlements) instead of continuous variables because much of the data were grouped according to certain distances. The three variables that involved human influence (distance to roads, paths, and inhabited places) were re-coded into two factors: short distance (<1500 m for roads, <1000 m for paths, <4000 m for settlements) or long distance (values greater than those listed above for each variable).

### Spatial analysis

An analysis was undertaken to determine whether a correlation existed between the number of dead birds found at each pylon and the distance between pylons. A marked point function was used [34], [35]. Marked point processes are used to determine whether there is a correlation between one value (in this case, the number of carcasses per pylon) and the distance between the pylons, or whether the cases of mortality are distributed in a random fashion [36]. As a result, we will be able to obtain the maximum distance between pylons were carcass distribution tends to cluster. So this distance between pylons may act as a diameter to represent areas where carcasses appears aggregated.

We used the function *K_mm_(d)* [37] to determine whether the carcass distribution tends to cluster (i.e. whether raptor mortality follows a ‘contagious’ pattern). In order to do this, we obtained the values that are taken by function *Kmm*(d) and, graphically, they were compared with random values (*random labelling*) at a 95% confidence interval. If the values of our function were higher than those obtained using random labelling, we considered this result to indicate a correlation between the mark (the mortality rate) and the distance between the pylons. Our interpretation of the analysis carried out is that, when mortality is concentrated, *d* can serve as the radius of the area in which the concentration occurs. Thus, by taking *d*, we can estimate the approximate size of the areas in which mortality tends to concentrate. The *K_mm_(d)* function is as follows:



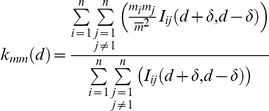
Where *d* is the distance between pylons, *m_i_* is the variable value in pylon *i*, δ is the interval calculation and I_ij_(d+δ,d-δ) is a product of density, and has a value of 1 if pylon *j* is within the area defined by two circles centred in pylon *i* and with a radius of *d*+δ,*d*-δ. This variable has a value of 0 in any other case [38].

We run two analyses. In the first one, the number of dead raptors, including eagles, is considered a mark. In the second one, only dead eagles are considered a mark. A total of 100 data replications per pylon were implemented for the study area. We carried out the calculation establishing a relationship between each pylon to those in a one km area

### Statistical analysis

In this study, each pylon was considered a sample unit. For each of the two dependent variables (the number of dead raptors including eagles and the number of large eagles found dead at each pylon), we fitted a generalised linear mixed model with a log-link function and a Poisson distribution [39]. Pylons included in the same power line were grouped by including the variable line as a random factor.

Throughout the analysis, models were simplified to eliminate statistically non-significant variables (α  = 5%). Once non-significant variables were removed, factor levels were grouped, if doing so did not change the model significantly, until the “minimal adequate model” [39] was reached. Models were adjusted for each of the two dependent variables to determine if mortality only depended on the structural characteristics of specific power lines (independent variables, Table 1), on environmental characteristics (independent variables, Table 2), or on a combination of both. The Akaikés information criterion (AIC) was used to determine the most parsimonious model in each case [40]. The statistical analyses were performed with software “R.2.8.0” (http://www.r-project.org/). Values are presented as mean±s.e.

## Results

### Mortality rate and distribution

A total of 952 electrocuted raptors were found, representing 14 different species. Of these, 929 (97.6%) were identified. We found that 16.6% (*n*  = 158) of all dead birds belonged to the genus *Aquila* (Table 3).

**Table 3:** Number of dead specimens by species and their corresponding threat level (Madroño et al. 2004).

Order	Scientific name	*n* (%)	Spanish Red List
Falconiformes	*Gyps fulvus*	30 (3.2)	Not evaluated
	*Aquila adalberti*	39 (4.2)	Endangered
	*Aquila fasciata*	54 (5.8)	Endangered
	*Aquila chrysaetos*	65 (7)	Near threatened
	*Circaetus gallicus*	68 (7.3)	Least concern
	*Hieraaetus pennatus*	2 (0.2)	Near threatened
	*Milvus milvus*	11 (1.2)	Endangered
	*Milvus migrans*	48 (5.2)	Near threatened
	*Buteo buteo*	367 (39.5)	Not evaluated
	*Accipiter gentilis*	23 (2.5)	Not evaluated
	*Falco tinnunculus*	29 (3.1)	Not evaluated
	*Falco naumanni*	2 (0.2)	Vulnerable
Strigiformes	*Bubo bubo*	189 (20.4)	Not evaluated
	*Asio otus*	2 (0.2)	Not evaluated
Undetermined	*-*	23	-
Total	*-*	952	-

Raptor mortality was caused by 610 pylons (10% of total). For these, the average number of electrocuted birds was 1.5±1 (1–7 range of electrocuted birds per pylon, *n*  = 610). For eagles, the average was 1.2±0.2 (1–6 range of electrocuted birds per pylon, *n*  = 133). Incidences of mortality for raptors were more homogenously distributed compared to eagles (Figure 1).

**Figure 2:**
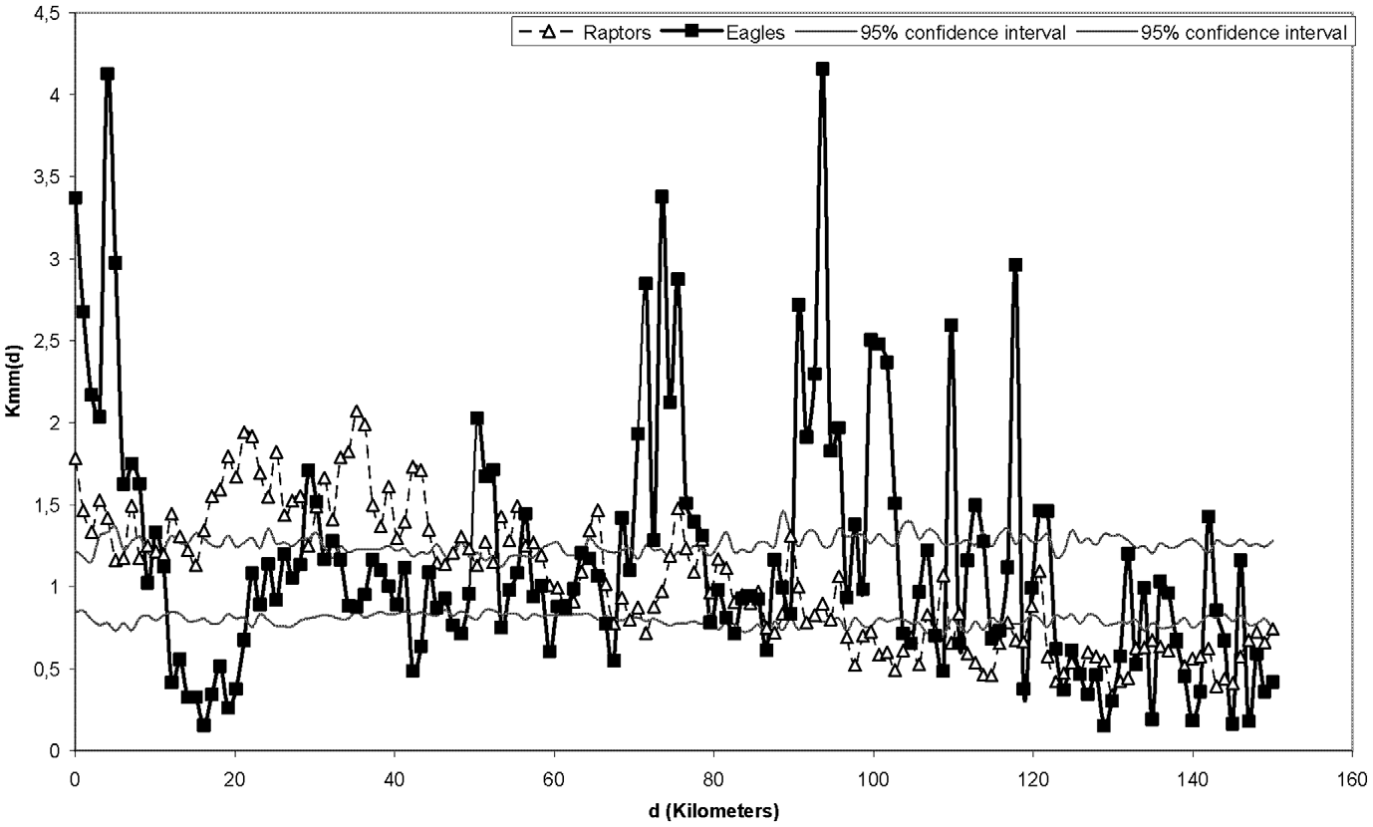
Values of *K_mm_*(d) for eagles and whole raptor species. On the x-axis distance (d) where function *K_mm_*(d) takes a value. If *K_mm_*(d) adopts values above the random distribution it implies mortality clustering phenomena within a area described through d.

The *K_mm_*(d) function shows whether the processes (mortality) tend to be spatially grouped (points above the 95% confidence interval) or occur randomly (within the confidence interval). The size of the areas in which mortality tends to cluster is defined approximately by the value of *d* when the values of the *K_mm_*(d) function are above the 95% confidence interval (Figure 2). Thus, in several areas, mortality does not follow a random pattern, but rather is spatially concentrated within those areas. In the case of eagles, (continuous line) incidences of mortality are concentrated within relatively small areas (7–10 km). For raptors in general, this phenomenon occurs in larger areas (20–40 km). This deviation compared to standard distribution reveals a ‘contagious’ mortality pattern for both groups.

### Factors related to electrocution

Raptor mortality caused by electrocution is a result of both environmental characteristics and the structure of the power lines (Table 4). When considering environmental factors, the number of electrocuted raptors increased as the number of prey animals increased (0.620±sd  = 0.060, *z*  = 10.384, *p* <0.001). A similar trend emerged for increasing bush cover (0.014±0.002; *z*  = 6.091; *p* <0.0001). Electrocutions decreased when distance to roads was above 1500 m (−0.340±0.112, *z*  = −3.027, *p*  = 0.002). Results for structural characteristics of the power lines indicated that electrocution rates increased when the number of insulators per phase decreased (-0.266±0.06, *z*  = −4.150, *p* <0.001). Electrocution rates also increased as the number of phases above the crossarm grew (0.238±0.070, *z*  = 3.418, *p* <0.001). When considering pylon function, results indicated that there was a significant difference in the number of electrocutions among the three types. Anchor-type pylons caused the highest number of electrocutions (1.446±0.171, *z*  = 8.477, *p* <0.001), followed by the special-type pylons (0.601±0.167, *z*  = 3.605, *p* <0.001). For crossarm more electrocutions were caused by pylons with cross-shaped and flat crossarms compared to other models (−0.641±0.191, *p* <0.001).

**Table 4:** Model selection for raptor and eagle mortality rates.

Dependent variable	Independent variables included	Minimal Adequate models	AIC
Raptor mortality rate	Structural+corrective measures	Function+model+mitigation measures+number of insulators+phases over the crossarm	3294.1
	Environmental	Prey abundance+dist roads+bush cover	3185
	Both	Prey abundance+dist roads+bush cover+function+ model +mitigation measures+number of insulators+phases over the crossarm	2980
Eagle mortality rate	Structural+corrective measures	Function+ model +number of insulators	1010.7
	Environmental	Slope+prey abundance+dist roads+ bush cover +tree cover	1161.5
	Both	Prey abundance+slope+ function+number of insulators	928.3

The independent variables initially included are specified, although model selection was based on the minimal adequate ones (after simplification).

The best-fitting model describing eagle mortality included structural and environment variables (Table 4). Eagle mortality rates differed among all pylon types. Anchor-type pylons caused the largest number of electrocutions (2.508±0.280, *z*  = 8.953, *p* <0.001) followed by special-type pylons (1.585±0.339, *z*  = 4.681, *p* <0.001). Mortality rates also increased depending on the number of insulators present (−0.515±0.110, *z*  = −4.659; *p* <0.0001), the slope near the pylons (0.044±0.021, z  = 2.029, p  = 0.043) and prey abundance (1.074±0.162, *z*  = 6.635, *p* <0.0001).

### Comparison of mortality between improved and unimproved power lines

**Figure 3:**
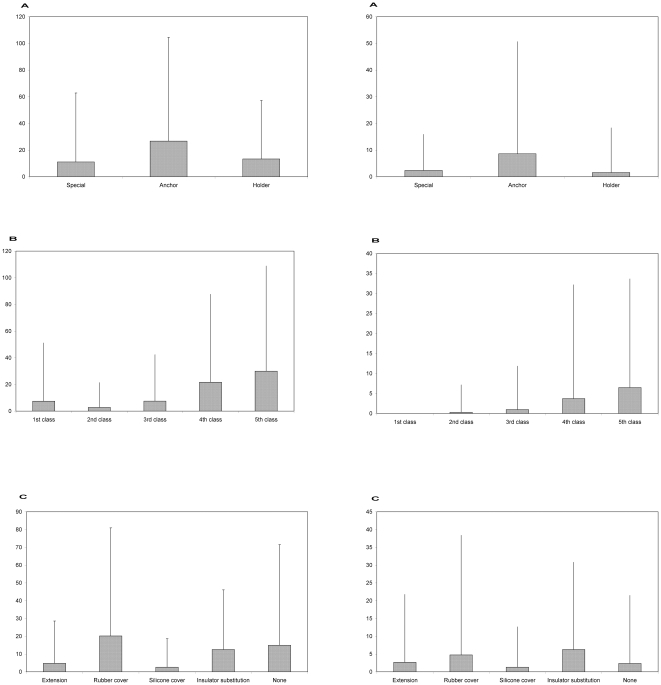
Electrocution rate of raptors (left column) and eagles (right column). Carcasses per 100 surveyed pylons for each pylon function (a), prey availability category (b) and type of mitigation measure (c).

Only pylons employing insulation extensions showed a lower raptor mortality rate (−1.195±0.519, *z*  = −2.303, *p*  = 0.0212), but not for eagles. No differences in mortality rate compared to uncorrected ones were observed for power lines employing other mitigation measures (Figure 3).

## Discussion

These results indicate that power lines cause a large number of deaths among many of the most threatened raptor species in Spain. Mortality rates found in this study are higher than others reported previously in different areas (2.6 eagle and 15.1 raptor carcasses per 100 pylons reviewed) [10], [11], [13], [16], [41], [42], but lower than results obtained in a nearby study area in 1998 (28.2/100 pylons reviewed) [13]. Three non-mutually exclusive hypotheses may explain the differences between these results. First, the types of power lines chosen for this study could potentially be more dangerous to raptors than those studied previously. This may be because, unlike the previous studies, 10% of the lines in the area *a priori* considered to be the most dangerous were sampled. A second possibility that our study area has a high density of raptors, being an important area for large eagle breeding [7], [20], [21] and in particular as a dispersal zone [19], [43], [44]. Since immature birds are more prone to electrocution [3], [10], [14], the electrocution rate rises when compared with the rates measured in the breeding areas. Finally, as a third possibility, the different carcass disappearance rates can modify the mortality rates found [45], [46]. Given the characteristics of this study in which the pylons were only checked once, in order to obtain a global mortality estimate, the data cannot be corrected for mortality rates by locality. When compared to the results obtained by [13] in a nearby area, differences may stem from the previously mentioned power line correction program [19]. This program has modified several pylons within this area. This hypothesis is supported by the lower electrocution rates of the eagles (2.6 electrocuted eagles per 100 pylons surveyed in our study compared to 3.3/100 pylons reviewed in [13]), which are prone to electrocution [42]. This is especially striking as Spanish imperial eagles and golden eagles have respectively increased [3], [47] and maintained their populations within the study area [21]. Meanwhile, Bonelli's eagle has suffered a slight decrease [20]. Thus, source-sink dynamics might be playing an important role, as has been documented in other species [48].

Analysis of the distribution of raptor electrocutions indicates that mortality is not constant across the entire study area. Mortality values, obtained using the *K_mm_*(d) function, suggests that incidences of electrocution tend to be concentrated in specific areas. Thus, raptor mortality rates are not evenly distributed throughout the study area, instead occurring within broad areas (with 20–40 km radius). This fact supports the idea that electrocutions are likely to be spatially related [11], [49]. This clustering effect might be due to concentrations of prey [32], as raptors' main prey species tend to gather in certain places [22], which may create areas with higher raptor densities. As a result, we should be careful to avoid making broad generalisations about bird mortality rates over large geographic areas [50].

For eagles, mortality rates occurred within smaller areas than those considered for raptors (with 7–10 km radius). A possible explanation is that, for eagles, deaths occur along a few closely-spaced power lines, possibly stemming from the fact that power lines in close proximity to each other are likely to be very similar [10], [11]. This cluster effect occurring for eagle electrocutions has been documented previously [3], [11], but this study expanded upon past research to determine that these deaths are linked to factors that make their deaths likely (e.g. habitat, slope, prey abundance, technical design) and which are particularly correlated. This fact is important when attempting to improve existing power lines to prevent future electrocutions.

For the two groups, both the pylon function and number of insulators affected mortality [11]. Moreover, in the case of the raptors, the number of phases above the crossarm affected mortality [14]. Furthermore, the design of the crossarm emerged as one of the most important factors affecting raptor electrocutions. In the case of the eagles, no type of crossarm was discovered to cause differences in mortality rates. This may stem from the larger wingspan of these birds, which is thought to contribute to their electrocution [42] and which facilitates their electrocution, irrespective of the design of the crossarm. Other structural characteristics of the pylons seem less important.

For both, eagles and raptors in general, prey abundance, among other factors, determined mortality rate, perhaps thus contributing to the grouping of deaths. For raptors in general, vegetation coverage also increased the chances of electrocution [10], [41], possibly because vegetation structure may affect prey availability and the predator foraging performance [5], [51]. Similarly, dominant pylons (i.e. tall structures in open areas) have been also shown to boost mortality [5]. In areas with greater human habitation and road banks, higher prey abundance was observed [22]. This suggests that more electrocutions occur in more humanized landscapes, despite the generally observed pattern [52], [53]. However, according our results we cannot state categorically the effect on different distance ranges, as suggested by other authors [52]. In addition, unlike raptors, the mortality rates for eagles increased with slope, possibly due to the habit of hunting from perches [54]. Previous studies have demonstrated that pylons located in dominant sites, surrounded by high slopes tend to produce higher electrocution rates [4], [11].

Many of the lines examined in the study area have similar designs, especially in the construction of the crossarm, likely related to standardisation by the power supplier. If we consider geographically both issues influencing electrocution rates, abundance of prey and pylon design, we are able to obtain the locations of highest mortality for raptors. Thus, the design of mortality monitoring programmes should take these factors into account, particularly in the case of eagles.

This study suggests that not all power line mitigation measures implemented have permanent effects in reducing raptor electrocutions. Here, we illustrated that mortality rates are higher in pylons that have only been insulated, in comparison with similar pylons. This may be provoked by the original insulation of the deadliest pylons. In this sense, after more than 10 years, the degradation process of insulation provokes higher electrocution rates compared with non-corrected pylons. Thus, our conclusion is that structural changes are also required [16]. This result contrast with those previously published [17], [10], [55], [56], which may be due to the conditions of the cable insulation carried out in the study area. Structural changes should focus on eliminating phases above the crossarm and increasing the distance between perch sites and wires, both of which influenced mortality for both groups of birds. For eagles, mortality rates were not influenced by any mitigation measure, so the only advisable strategy is the implementation of structural modifications (changing crossarm and increasing the length of the string of insulators) and not only including extensions.

Importantly, results from this study suggests that the insulation of exposed conductors in ground-wired pylons, as it has been developed in this area, is a practice that is inefficient in long-term raptor electrocution rate reduction (see [55], [56] in contrast). This low efficiency rate may in part be due to the time that has elapsed (on average 15 years) since this insulation was installed. Since then, very little or no maintenance has been carried out, despite this being an area with a very harsh climate.

Eagle deaths seemed to concentrate around a small group of power lines that were located near a large rabbit population. As crossarm design did not affect these electrocution incidences (all caused similar mortality rates, but see [10], [11]), when designing monitoring programmes, it would be useful to check all the power lines in the area. Thus, a new priority could be to concentrate mitigation measures on power lines causing the highest numbers of electrocutions and those in the immediate proximity. Our results suggests that mitigation measures must be implemented along the entire line. Other authors suggested, for certain circumstances, a “preferred pylon” approach [11], [49]. However, although more research is needed and solutions must be developed case-by-case, we consider our results might be applicable to any other ground-wired power network.

## Supporting Information

Figure S1 Flat crossarm in an anchor pylon with three insulators and one phase over the crossarm.
(TIF)

Figure S2 Cross-shaped crossarm in a holder pylon with pin-type insulators and three phases over the crossarm.
(TIF)

Figure S3 Vault crossarm in a special pylon (derivation) with two insulators and no phases over the crossarm.
(TIF)

Figure S4 Lattice vault crossarm in an anchor pylon with seven insulators and no phases over the crossarm.
(TIF)

Figure S5 Three level crossarm in a holder pylon, silicone covers as mitigation measures, three insulators and no phases over the crossarm.
(TIF)

## References

[1] Bevanger K (1998). Biological and conservation aspects of bird mortality caused by electricity power lines: a review.. Biol Conserv.

[2] Real J, Grande JM, Mañosa S, Sánchez-Zapata JA (2001). Causes of death in different areas for Bonelli's Eagle *Hieraaetus fasciatus* in Spain.. Bird Study.

[3] Gonzalez LM, Margalida A, Mañosa S, Sánchez R, Oria J (2007). Causes and spatio-temporal variations of non-natural mortality in the Vulnerable Spanish imperial eagle *Aquila adalberti* during a recovery period.. Oryx.

[4] Lehman RN, Kennedy PL, Savidge JA (2007). The state of the art in raptor electrocution research: A global review.. Biol Conserv.

[5] Sergio F, Marchesi L, Pedrini P, Ferrer M, Penteriani V (2004). Electrocution alters the distribution and density of a top predator, the eagle owl *Bubo bubo*.. J Appl Ecol.

[6] Soutullo A, López-López P, Urios V (2008). Incorporating spatial structure and stochasticity in endangered Bonelli's eagle's population models: Implications for conservation and management.. Biol Conserv.

[7] Ortega E, Mañosa S, Margalida S, Sanchez R, Oria J (2009). A demographic description of the recovery of the Vulnerable Spanish imperial eagle *Aquila adalberti*.. Oryx.

[8] Penteriani V, Otalora F, Ferrer M (2005). Floater survival affects population persistence. The role of prey availability and environmental stochasticity.. Oikos.

[9] BirdLife International (2004). Birds in Europe: Population Estimates, Trends and Conservation Status.BirdLife Conservation Series 12..

[10] Janss GFE, Ferrer M (2001). Avian electrocution mortality in relation to pole design and adjacent habitat in Spain.. Bird Conserv Int.

[11] Mañosa S (2001). Strategies to identify dangerous electricity pylons for birds.. Biodiv Conserv.

[12] Janss GFE, Ferrer M (1999). Mitigation of raptor electrocution on steel power poles.. Wildl Soc Bull.

[13] Guzman J, Castaño JP (1998). Electrocución de rapaces en líneas eléctricas de distribución en Sierra Morena oriental y Campo de Montiel.. Ardeola.

[14] Harness RE, Wilson KR (2001). Electric-utility structures associated with raptor electrocutions in rural areas.. Wildl Soc Bull.

[15] Mojica EK, Watts BD, Paul JT, Voss ST, Pottie, J (2009). Factors contributing to bald eagle electrocutions and line collisions on Aberdeen Proving Ground, Maryland.. J Raptor Res.

[16] Tintó A, Real J, Mañosa S (2010). Predicting and correcting electrocution of birds in Mediterranean areas.. J Wildl Manage.

[17] López-López P, Ferrer M, Madero A, Casado E, McGrady M (2011). Solving man-induced large-scale conservation problems: the Spanish imperial eagle and power lines.. PLoS ONE.

[18] LIFE Unit (2010). LIFE project database.. http://ec.europa.eu/environment/life/.

[19] MMA (2001). Estrategia Nacional para la Conservacion del Aguila imperial iberica..

[20] Del Moral JC (2006). El águila perdicera en España. Población reproductora en 2005 y método de censo..

[21] Del Moral JC (2009). El águila real en España.Población reproductora en 2008 y método de censo..

[22] Delibes-Mateos M, Ferreras P, Villafuerte R (2008). Rabbit populations and game management: the situation after 15 years of rabbit haemorrhagic disease in central-southern Spain.. Biodiv Conserv.

[23] Villafuerte R, Calvete C, Blanco JC, Lucientes J (1995). Incidence of viral haemorragic disease in wild rabbit populations in Spain.. Mammalia.

[24] Madroño A, González C, Atienza JC (2004). Libro Rojo de las Aves de España..

[25] Marti R, Del Moral JC (2003). Atlas de las aves reproductoras de España..

[26] Dwyer JF, Mannan RW (2007). Preventing raptor electrocution in an urban environment.. J Raptor Res.

[27] Palomino D, Carrascal LM (2007). Habitat associations of a raptor community in a mosaic landscape of Central Spain under urban development.. Lands Urban Plan.

[28] Hager SB (2009). Human-related mortality sources to urban raptors.. J Raptor Res.

[29] Negro JJ, Ferrer M (1995). Mitigating measures to reduce electrocution of birds on power lines: a comment on Bevanger's review.. Ibis.

[30] MMA (2004). Mapa Forestal de España, 1∶50.000, Albacete province..

[31] MMA (2004). Mapa Forestal de España, 1∶50.000, Ciudad Real province..

[32] Rubolini D, Bassi E, Bogliani G, Galeotti P, Garavaglia R (2001). Eagle Owl *Bubo bubo* and power line interactions in the Italian Alps.. Bird Conserv Int.

[33] Jenness J (2004). Nearest features extension for ArcView 3.x, v. 3.8a.. http://www.jennessent.com/arcview/nearest_features.htm.

[34] Montes F, Barbeito I, Rubio A, Cañellas I (2008). Evaluating height structure in Scots pine using marked point processes.. Can J Forest Res.

[35] Penttinen A Stoyan D, Hentonnen HD (1992). Marked point processes in forest statistics.. Forest Sci.

[36] Beisbart C, Kerscher M, Mecke K (2002). Mark correlations: relating physical properties to spatial distributions.. Lect Notes Phys.

[37] Stoyan D, Stoyan H (1994). Fractal, random shapes and point fields..

[38] Wiegand T, Moloney KA (2004). Ring, circles and null-models for point pattern analysis in ecology.. Oikos.

[39] Crawley MJ (2007). The R Book..

[40] Burnham KP, Anderson DR (2002). Model selection and inference: a practical information-theoretic approach. 2nd ed..

[41] Ferrer M, De La Riva M, Castroviejo J (1991). Electrocution of raptors on power lines in southwestern Spain.. J Field Ornithol.

[42] Janss GFE (2000). Avian mortality from power lines: a morphologic approach of a species-specific mortality.. Biol Conserv.

[43] Cadahia L, López-López P, Urios V, Negro JJ (2010). Satellite telemetry reveals individual variation in juvenile Bonelli's eagle dispersal areas.. Eur J Wildl Res.

[44] González LM, Oria J, Margalida A, Sánchez R, Prada L (2006). Effective natal dispersal and age of maturity in the threatened Spanish Imperial Eagle *Aquila adalberti*: conservation implications.. Bird Study.

[45] Lehman RN, Savidge JA, Kennedy PL, Harness RE (2010). Raptor electrocution rates for a utility in the intermountain Western United States.. J Wildl Manage.

[46] Ponce C, Alonso JC, Argandoña G, García Fernández A, Carrasco M (2010). Carcass removal by scavengers and search accuracy affect bird mortality estimates at power lines.. Anim Conserv.

[47] González LM, Oria J, Sánchez R, Margalida A, Aranda A (2008). Status and habitat changes in the endangered Spanish Imperial Eagle *Aquila adalberti* population during 1974-2004: implications for its recovery.. Bird Conserv Int.

[48] Schaub M, Aebischer S, Gimenez O, Berger S, Arlettaz R (2010). Massive immigration balances high anthropogenic mortality in a stable eagle owl population: Lessons for conservation.. Biol Conserv.

[49] Williams RD, Colson EW (1989). Raptor association with linear rights-of-way. Western Raptor Management Symposium and Workshop..

[50] Moleón M, Bautista J, Garrido JR, Martín-Jaramillo J, Ávila E (2007). Correcting power lines in dispersal areas of Bonelli's eagles: potential positive effects on the raptor community.. Ardeola.

[51] Fernández N (2005). Spatial patterns in European rabbit abundance after a population collapse.. Lands Ecol.

[52] Palomino D, Carrascal LM (2007). Threshold distances to nearby cities and roads influence the bird community of a mosaic landscape.. Biol Conserv.

[53] Bautista LM, García JT, Calmaestra R, Palacín C, Martín CA (2004). Effect of weekend road traffic on the use of space by raptors.. Conserv Biol.

[54] Ferguson-Lees J, Christie DA (2001). Raptors of the World..

[55] Avian Power Line Interaction Comitee (2006). Suggested Practices for Avian Protection on Power Lines: The State of the Art in 2006..

[56] Haas D, Nipkow M, Fiedler G, Schneider R, Haas W (2005). Protecting birds from powerlines..

