# Supplementary figures and images for: Minimising Mortality in Endangered Raptors Due to Power Lines: The Importance of Spatial Aggregation to Optimize the Application of Mitigation Measures
Source: PLoS One. 2011 Nov 28;6(11):e28212. doi: 10.1371/journal.pone.0028212 (PMC3225394; doi:10.1371/journal.pone.0028212)

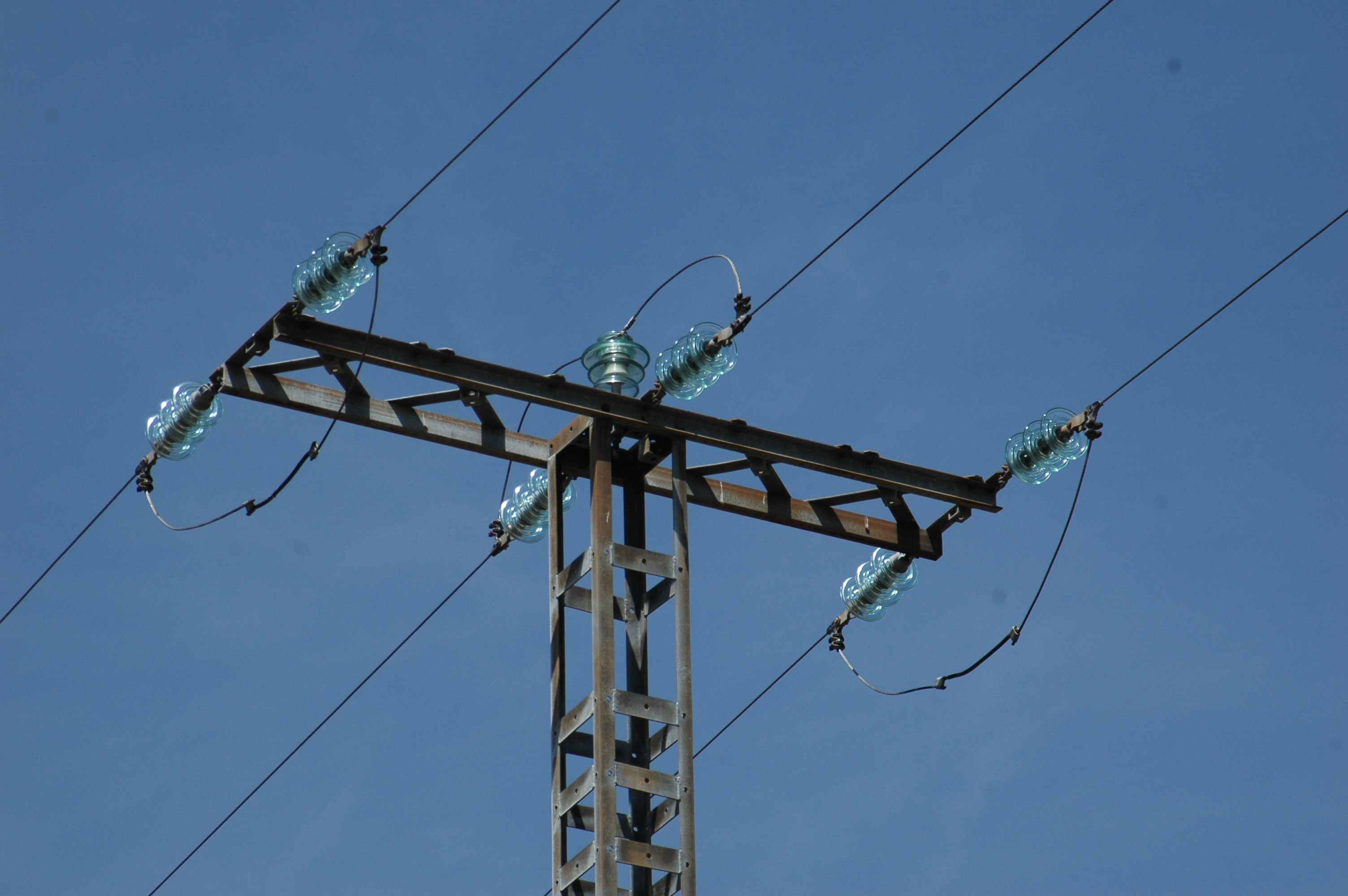

Supplement: Figure S1 — Flat crossarm in an anchor pylon with three insulators and one phase over the crossarm. (TIF) [file pone.0028212.s001.tif]

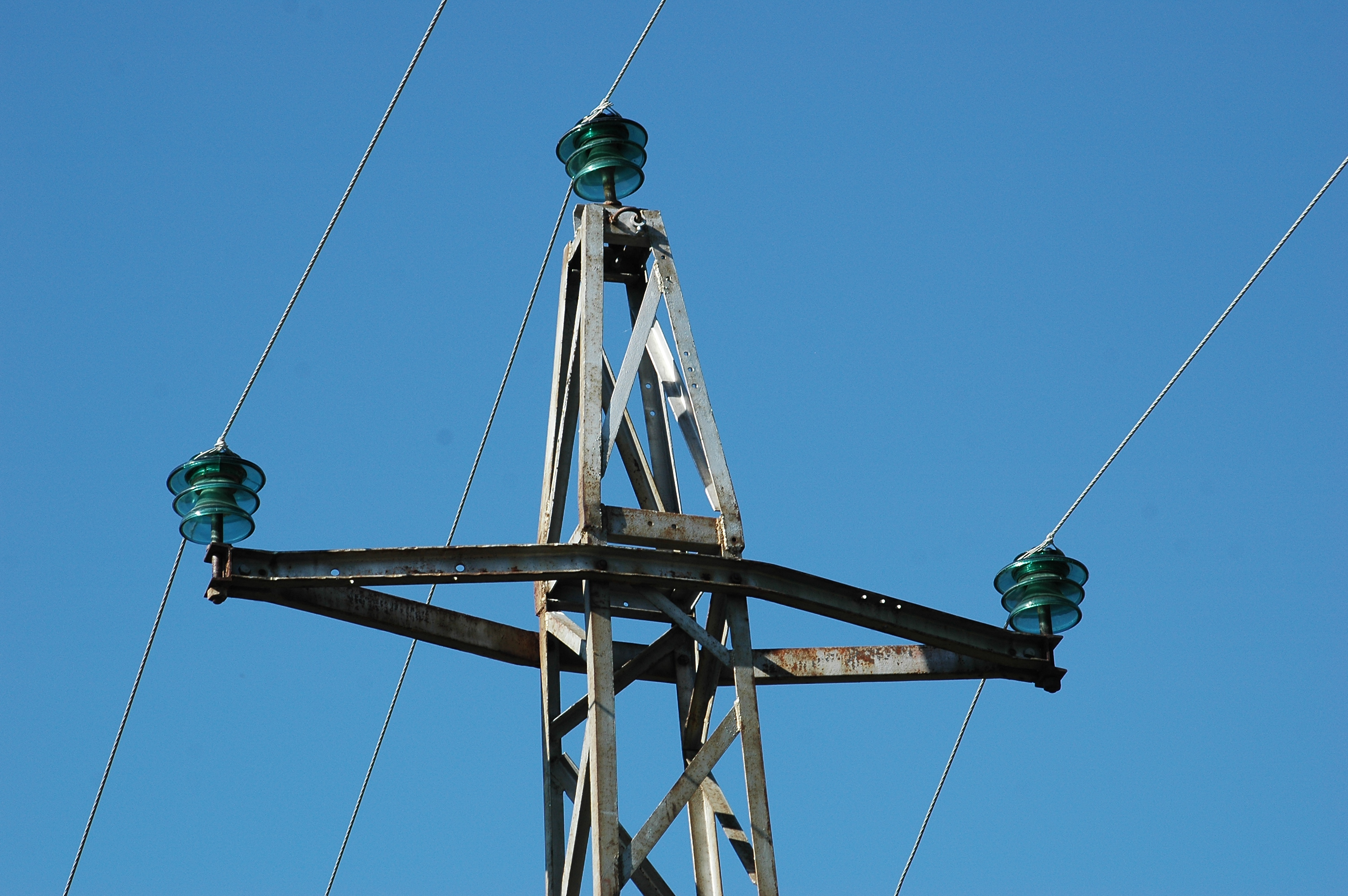

Supplement: Figure S2 — Cross-shaped crossarm in a holder pylon with pin-type insulators and three phases over the crossarm. (TIF) [file pone.0028212.s002.tif]

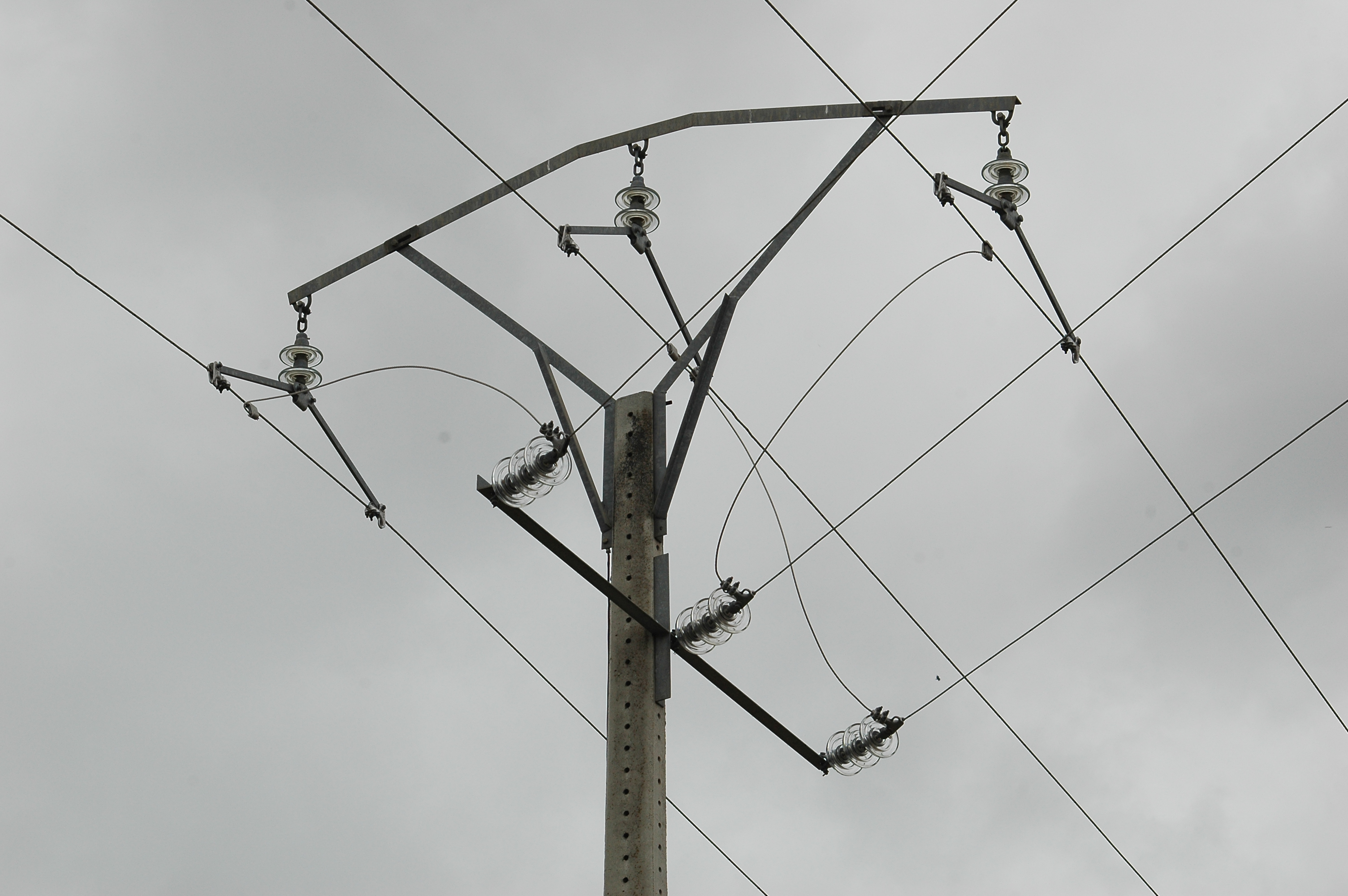

Supplement: Figure S3 — Vault crossarm in a special pylon (derivation) with two insulators and no phases over the crossarm. (TIF) [file pone.0028212.s003.tif]

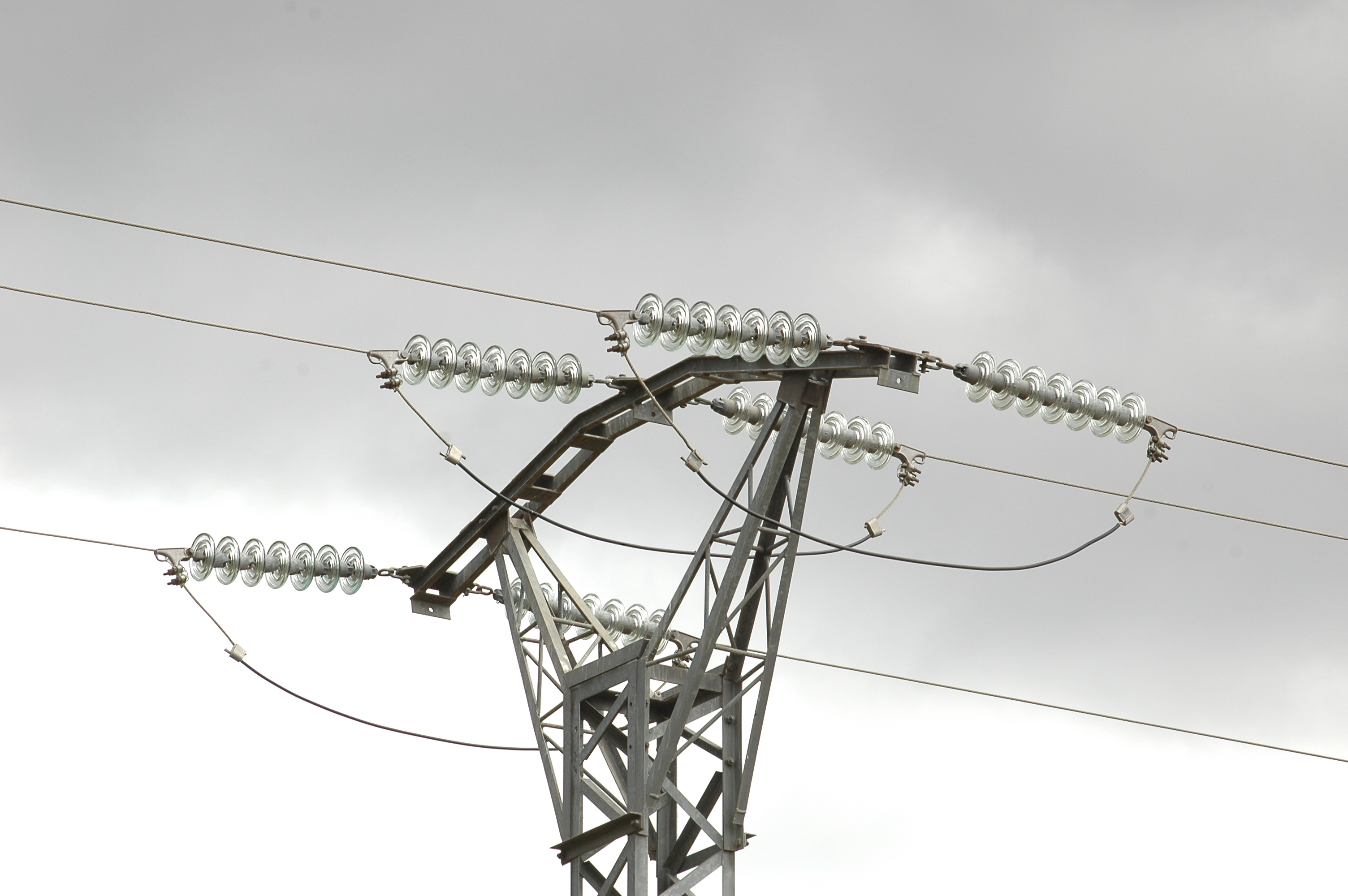

Supplement: Figure S4 — Lattice vault crossarm in an anchor pylon with seven insulators and no phases over the crossarm. (TIF) [file pone.0028212.s004.tif]

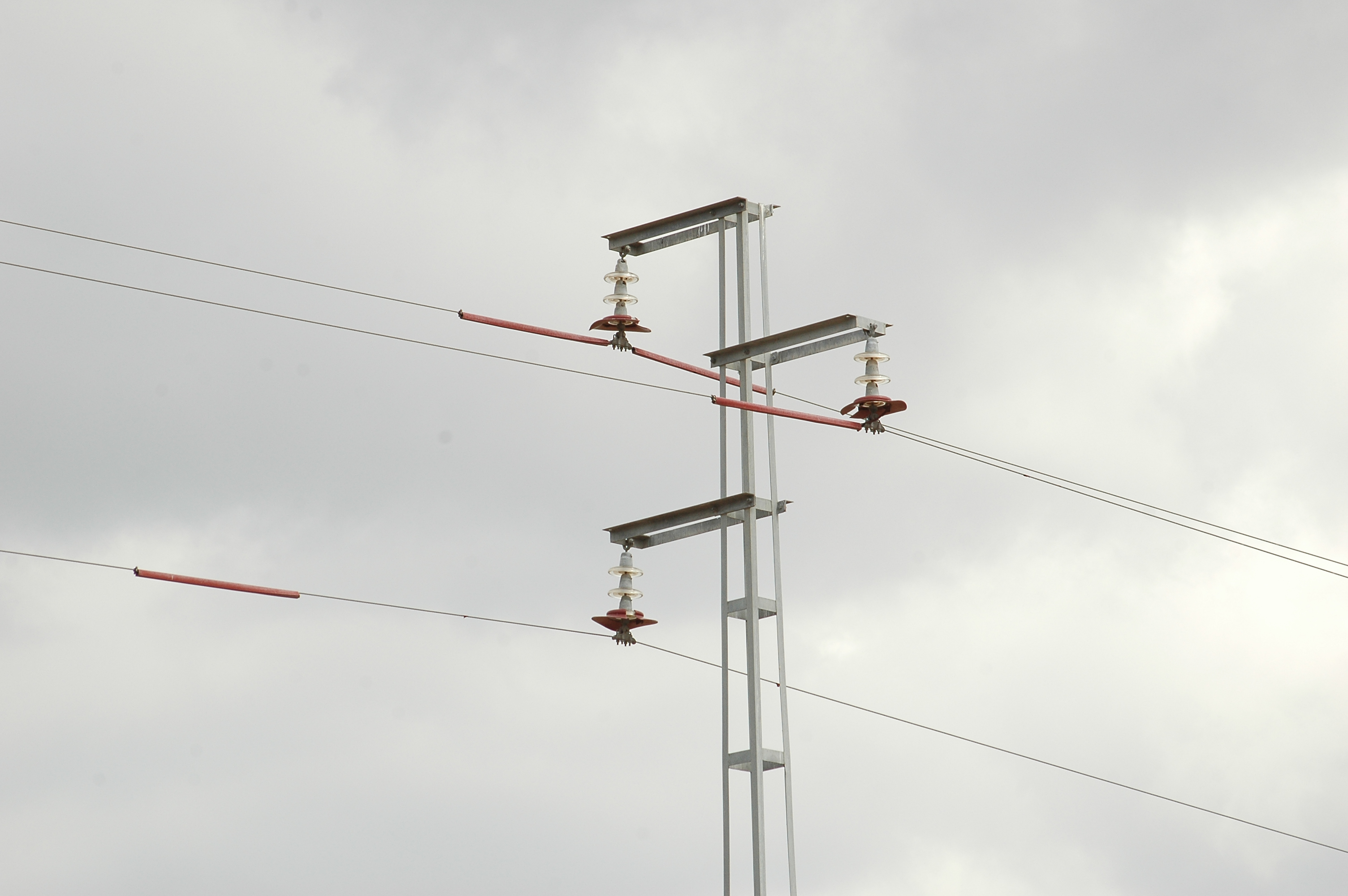

Supplement: Figure S5 — Three level crossarm in a holder pylon, silicone covers as mitigation measures, three insulators and no phases over the crossarm. (TIF) [file pone.0028212.s005.tif]
